# Identification of New Mutations at the PCNA Subunit Interface that Block Translesion Synthesis

**DOI:** 10.1371/journal.pone.0157023

**Published:** 2016-06-03

**Authors:** Christine M. Kondratick, Elizabeth M. Boehm, Lynne M. Dieckman, Kyle T. Powers, Julio C. Sanchez, Samuel R. Mueting, M. Todd Washington

**Affiliations:** 1 Department of Biochemistry, Carver College of Medicine, University of Iowa, Iowa City, Iowa, 52242, United States of America; 2 Department of Chemistry, Creighton University, Omaha, Nebraska, 68178, United States of America; University of Miami Miller School of Medicine, UNITED STATES

## Abstract

Proliferating cell nuclear antigen (PCNA) plays an essential role in DNA replication and repair by interacting with a large number of proteins involved in these processes. Two amino acid substitutions in PCNA, both located at the subunit interface, have previously been shown to block translesion synthesis (TLS), a pathway for bypassing DNA damage during replication. To better understand the role of the subunit interface in TLS, we used random mutagenesis to generate a set of 33 PCNA mutants with substitutions at the subunit interface. We assayed the full set of mutants for viability and sensitivity to ultraviolet (UV) radiation. We then selected a subset of 17 mutants and measured their rates of cell growth, spontaneous mutagenesis, and UV-induced mutagenesis. All except three of these 17 mutants were partially or completely defective in induced mutagenesis, which indicates a partial or complete loss of TLS. These results demonstrate that the integrity of the subunit interface of PCNA is essential for efficient TLS and that even conservative substitutions have the potential to disrupt this process.

## Introduction

Proliferating cell nuclear antigen (PCNA) is a protein that is essential for many DNA metabolic processes including DNA replication, base excision repair, nucleotide excision repair, mismatch repair, and recombination [1–4]. PCNA is a ring-shaped homo-trimer that acts as a sliding clamp that encircles DNA forming a scaffold for binding numerous proteins involved in DNA metabolism. Many of these proteins—which include DNA polymerases, mismatch repair proteins, endonucleases, and others—bind PCNA via conserved PCNA-interacting protein (PIP) motifs that dock in a cleft in the front face of the PCNA ring [1,2,4].

PCNA is required for translesion synthesis (TLS), which is a pathway for bypassing DNA damage during replication [5–12]. During TLS, PCNA is mono-ubiquitylated at Lys-164 in response to replication fork stalling [13]. This is necessary for the switch between the stalled replicative polymerase and a specialized TLS polymerase [14]. Depending on the type of DNA lesion and the specific polymerase utilized, TLS can be either error-free or error-prone. Error-free TLS, which does not lead to mutations, is best exemplified by the bypass of thymine dimers by DNA polymerase eta (pol η) [15]. Defects in error-free TLS lead to an increase in the rate of damage-induced mutagenesis [16]. In humans, such defects are responsible for the variant form of the skin cancer-prone disorder xeroderma pigmentosum (XPV) [17,18]. Error-prone TLS, which does lead to mutations, usually involves two TLS polymerases working in concert, such as Rev1 and DNA polymerase zeta (pol ζ). Defects in error-prone TLS lead to a decrease in the rate of damage-induced mutagenesis [19].

Two amino acid substitutions in PCNA (E113G and G178S) have been identified that disrupt translesion synthesis in yeast without interfering with normal DNA replication or other DNA repair pathways [20–22]. The corresponding PCNA mutant proteins have been characterized biochemically and their X-ray crystal structures have been determined [23–26]. Both of these substitutions are at the subunit interface of PCNA between adjacent monomers, and both have similar structural alterations in the β strands and loops at the interface. These findings supported the view that the integrity of the subunit interface is critical to the ability of PCNA to function in TLS. Analyses of these structures, however, have suggested that these structural changes at the subunit interface appear to be peculiar to these specific substitutions. Thus it is unclear whether additional substitutions at the subunit interface will compromise the ability of PCNA to support TLS.

The subunit interface of PCNA is comprised of two anti-parallel β strands: β strand I_1_ in domain 1 of one subunit and β strand D_2_ in domain 2 of the adjacent subunit. To better understand the role of the subunit interface in translesion synthesis and in other DNA metabolic processes, we used random mutagenesis to generate a series of PCNA mutant proteins with substitutions in either of these two β strands. We created a set of 33 PCNA mutants that we assayed for viability and sensitivity to ultraviolet (UV) radiation using a spotting assay. We then selected a subset of 17 PCNA mutants that displayed the full range of phenotypes from the spotting assay and carried out quantitative assays for cell growth, sensitivity to UV radiation, spontaneous mutagenesis, and UV-induced mutagenesis. Of these, 14 were partially or completely defective in UV-induced mutagenesis indicating a partial or complete loss of error-prone TLS. These results demonstrate that the integrity of the PCNA subunit interface is essential for efficient TLS. Further biochemical characterization and structural analyses of these new PCNA mutant proteins should provide important new insights into TLS and how it is regulated by PCNA.

## Materials and Methods

### Plasmids and proteins

To carry out random mutagenesis of five residues (positions 111 to 115) along β strand I_1_ and of five residues (positions 177 to 181) along β strand D_2_, we obtained ten oligonucleotides from Integrated DNA Technologies (Coralville, IA). Each of these ten oligonucleotides had all four nucleotides randomly incorporated into all three positions of a single codon. The five oligonucleotides with random incorporations in the sequence corresponding to residues on β strand I_1_ were mixed together, and all of the random mutagenesis of this strand was done in a single reaction using the QuikChange Multi Site-Directed Mutagenesis Kit (Agilent Technologies). Similarly, the five oligonucleotides with random incorporations in the sequence corresponding to residues on β strand D_2_ were mixed, and all of the random mutagenesis of this strand was done in another single reaction. The template for both mutagenesis reactions was pKW371, which contains the wild type *POL30* gene (which encodes PCNA) with additional 500 base pairs of genomic DNA on the 5' end of the gene inserted into the pRS315 (*LEU2*) yeast shuttle vector. We sequenced 96 plasmids from these two mutagenesis reactions and obtained 15 plasmids harboring genes encoding PCNA mutant proteins with unique substitutions in strand I_1_ and 18 plasmids harboring genes encoding PCNA mutant proteins with unique substitutions in strand D_2_. These 33 plasmids were given designations of pKW488 to pKW520 (**Table 1**) and were used in assays for cell growth, sensitivity to ultraviolet (UV) radiation, and UV-induced mutagenesis. Other plasmids used in these assays include pKW371, pKW372, pKW373, and pKW487, which encode the wild type, the G178S mutant, the K164R mutant, and the E113G mutant PCNA proteins, respectively, in the same pRS315 yeast shuttle vector.

**Table 1:** PCNA random mutant proteins.

Substitution	Plasmid	Growth	UV resistance
I111E	pKW488	++	+
I111L	pKW489	+	ND
I111P	pKW490	++	+
I111R	pKW491	+	ND
I111V	pKW492	+	ND
A112E	pKW493	++	+
A112G	pKW494	++	++
A112R	pKW495	++	++
E113A	pKW496	++	+
Y114A	pKW497	+++	+
Y114F	pKW498	+++	+
S115G	pKW499	+	ND
S115E	pKW500	+	ND
S115N	pKW501	+++	++
S115V	pKW502	+++	++
S177E	pKW503	+	ND
S177G	pKW504	+	ND
S177L	pKW505	+	ND
S177V	pKW506	+	ND
S177Q/G178A	pKW507	+	ND
G178L	pKW508	+	ND
G178M	pKW509	++	++
S179A	pKW510	++	++
S179P	pKW511	++	++
S179R	pKW512	++	++
S179T	pKW513	+++	++
S179V	pKW514	+	ND
V180A	pKW515	+++	+
V180R	pKW516	+	ND
I181G	pKW517	+	ND
I181P	pKW518	+	ND
I181R	pKW519	+++	-
I181V	pKW520	++	+

ND = not determined; +++ indicates similar growth and UV resistance to wild type; ++ indicates slightly reduced growth compared to wild type; + indicates dramatically reduced growth compared to wild type (especially at the 10^8^ cells/ml and 50 J/m^2^);–indicates no growth.

### Assays for cell growth, UV sensitivity, and mutagenesis

The yeast shuttle vectors (*LEU2*) containing the genes for wild type PCNA (pKW371), the K164R mutant PCNA protein (pKW373), and the other 33 mutant proteins (pKW488 to pKW520) were transformed into yeast strain EMY74.7 with a *POL30* gene deletion. This strain harbored the plasmid pKW532 (*URA3*) containing the wild type *POL30* gene under control of its native promoter as described [27]. Following counter-selection with 5’-fluoroorotic acid, serial dilutions of yeast culture containing 10^8^, 10^7^, 10^6^, 10^5^, 10^4^, and 10^3^ cells per mL were spotted on synthetic complete media lacking leucine. These cells were exposed to UV radiation (50 J/m^2^ or 100 J/m^2^) and incubated in the dark for 3 to 4 days. Growth rates for these strains were determined by inoculating 1 x 10^5^ yeast cells in 100 mL of liquid synthetic complete media lacking leucine at 30°C and measuring optical density at 600 nm. These strains were assayed for UV resistance and UV-induced mutagenesis. For UV resistance, dilutions of cells (10^3^, 10^4^, 10^5^, 10^6^, 10^7^, and 10^8^ cells per ml) were plated on synthetic complete media lacking leucine for a given UV dose of 0, 25, 50, 75, 100, & 150 J/m^2^. Two plates of cells were exposed to each dose of UV radiation and incubated at 30°C in the dark for 3 to 4 days prior to counting and averaging the number of surviving cells. The percentage of surviving cells at each UV dose was determined by dividing the number of colonies at a given UV dose by the number of colonies at 0 J/m^2^. For UV-induced mutagenesis, yeast cells were plated in duplicate at 10^8^ cells per ml on synthetic complete media lacking arginine and containing canavanine prior to UV irradiation. The number of colonies that grew on canavanine-containing plates were counted and normalized to the number of colonies that grew on plates lacking canavanine at the same UV dose. The spontaneous mutagenesis rate is determine by the number of colonies that grow on media containing canavanine at 0 J/m^2^.

## Results

**Fig 1:**
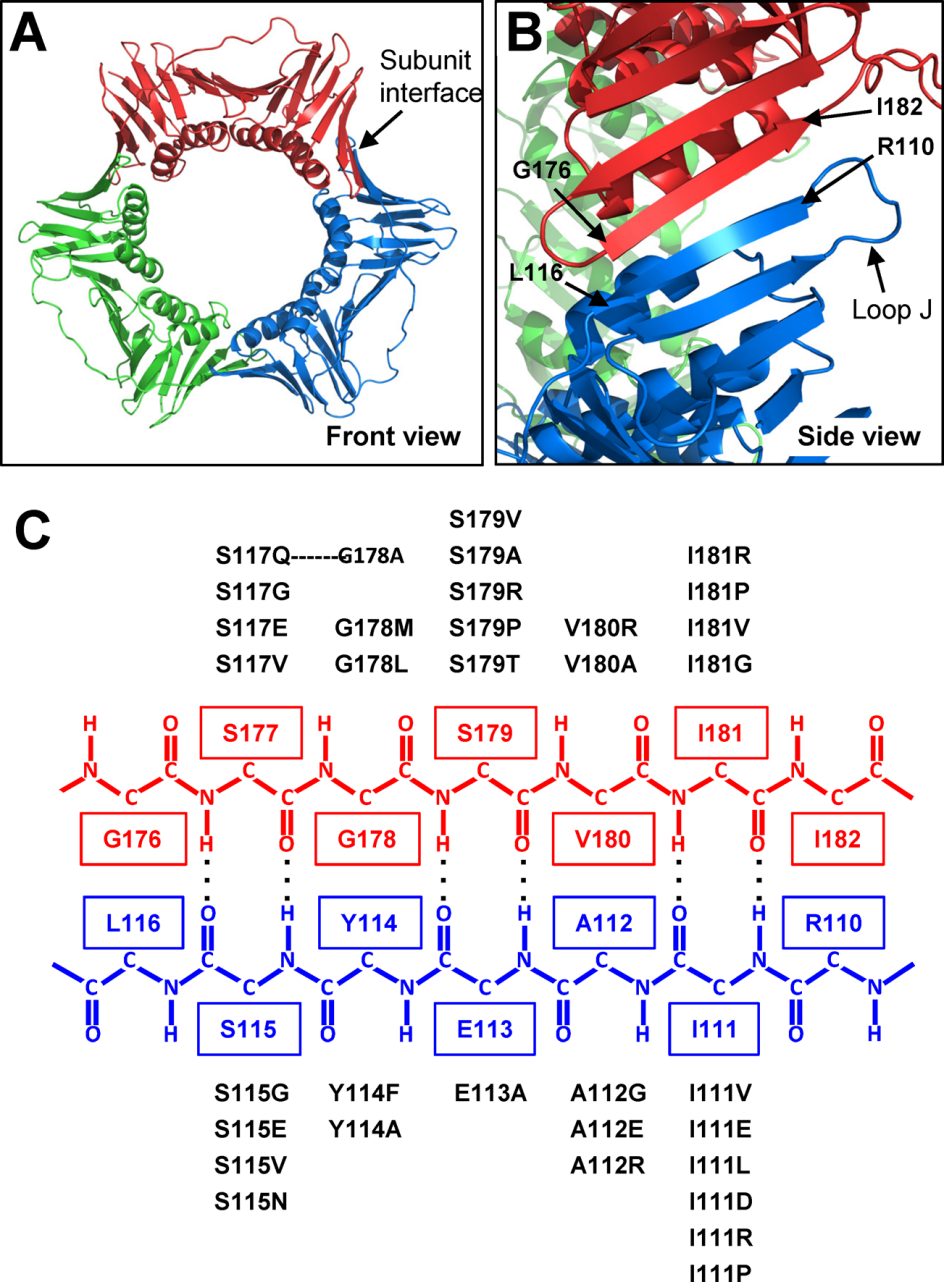
Random amino acid substitutions of the PCNA subunit interface. (**A**) Front view of the structure of PCNA with the location of the subunit interface highlighted. (**B**) Side view of the structure of PCNA with the subunit interface shown. (**C**) A schematic of the interface of PCNA is shown with the proper hydrogen bonds indicated. The wild type residues in β strand I_1_ are shown in blue with the substitutions generated at each residue listed below each wild type residue. The wild type residues in β strand D_2_ are shown in red with the substitutions generated at each residue listed above each wild type residue.

The subunit interface of PCNA is comprised of two anti-parallel β strands: strand I_1_ (residues 109 to 117) in domain 1 of one subunit and strand D_2_ (residues 175 to 183) in domain 2 of the adjacent subunit. Substitutions of two residues within these β strands have been shown to block translesion synthesis [20,21]. To better understand the role of the subunit interface in causing this TLS block, we have used random mutagenesis to generate a series of PCNA mutants with substitutions in these two strands. In strand I_1_, we made substitutions of residues 111 to 115, and in strand D_2_, we made substitutions of residues 177 to 181. We generated a set of 33 PCNA mutants with 15 having substitutions in strand I_1_ and 18 having substitutions in strand D_2_ (Fig 1).

### Growth and sensitivity to UV radiation of the full set of random mutants

**Fig 2:**
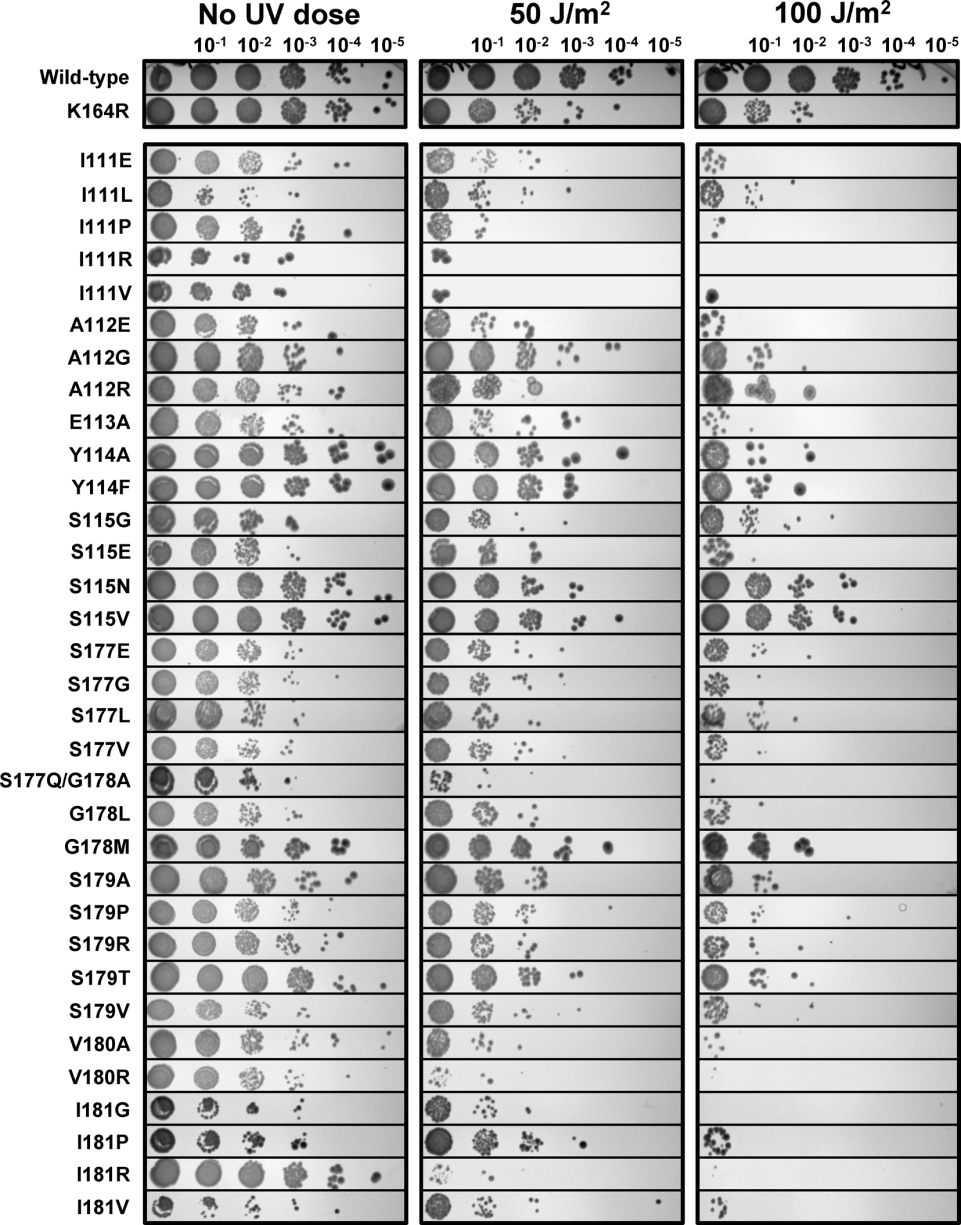
Viability and UV sensitivity of all 33 PCNA mutants by the spotting assay. Yeast cultures were grown expressing either wild type PCNA or mutant PCNA. These strains were spotted at varying concentrations from 10^3^−10^8^ cells per ml. The cells were stamped without exposure to UV, with exposure to 50 J/m^2^ UV, and with exposure to 100 J/m^2^ UV.

We initially assayed the entire set of 33 PCNA mutants for cell growth and sensitivity to UV radiation by spotting serial dilutions of yeast cultures on plates and exposing then to increasing doses of UV radiation (Fig 2, Table 1). All of the PCNA mutants produced for this study supported cell viability. Comparison of the spots in the absence of UV exposure, however, showed that many of these PCNA mutants display minor or major defects in cell growth. Some PCNA mutants, such as Y114A, S115N, and others showed similar levels of cell growth compared to wild type PCNA. PCNA mutants such as I111E, A112E, and others showed minor defects in cell growth compared to wild type PCNA. PCNA mutants such as I111L, S115G, and others showed major defects in cell growth.

Sensitivity to UV radiation is often used as a measure of the ability of cells to carry out DNA repair or bypass of UV-induced DNA lesions. Comparison of the spots in the presence of various doses of UV radiation showed that some of the PCNA mutants displayed an increased sensitivity to UV radiation compared to the wild type. As a control, we also examined the UV sensitivity of the K164R PCNA mutant, which produces a protein that cannot be ubiquitylated and is defective in DNA damage bypass. In strains where we observed normal cell growth, clear sensitivities to UV radiation could be observed compared to wild type cells. These strains included Y114A, S115N, and others. In strains where we observed a minor defect in cell growth, it was still possible to observe clear sensitivities to UV radiation. These strains included I111E, A112E, and others. The phenotypes of these two classes of strains are consistent with defects in translesion synthesis as exemplified by the previously characterizes E113G and G178S PCNA mutants [20–22]. In strains where we observed a major defect in cell growth, it was difficult to conclude anything about their sensitivities to UV radiation from this assay. Overall, we concluded that only the S115N and S115V strains had nearly wild type levels of both cell growth and UV sensitivity.

### Growth rate, sensitivity to UV radiation, and UV-induced mutagenesis of a subset of random mutants

We selected a subset of 17 PCNA mutants for more detailed, quantitative analyses of their growth rate, UV sensitivities, and UV-induced mutagenesis. This subset was chosen to represent the full range of phenotypes that we observed with the spotting assay including those that had no defects in cell growth (Y114A, Y114F, S115N, S115V, S179T, V180A, and I181R), minor defects in cell growth (A112G, S177G, S179A, S179R, and G178M), and major defects in cell growth (S115E, S115G, S177L, S177V, G178L) as well as minor defects in UV sensitivity (A112G, S115N, S115V, G178M, S179A) and major defects in UV sensitivity (Y114A, Y114F, S115G, S115E, S177G, S177L, S177V, G178L, S179R, S179T, V180A, and I181R).

**Fig 3:**
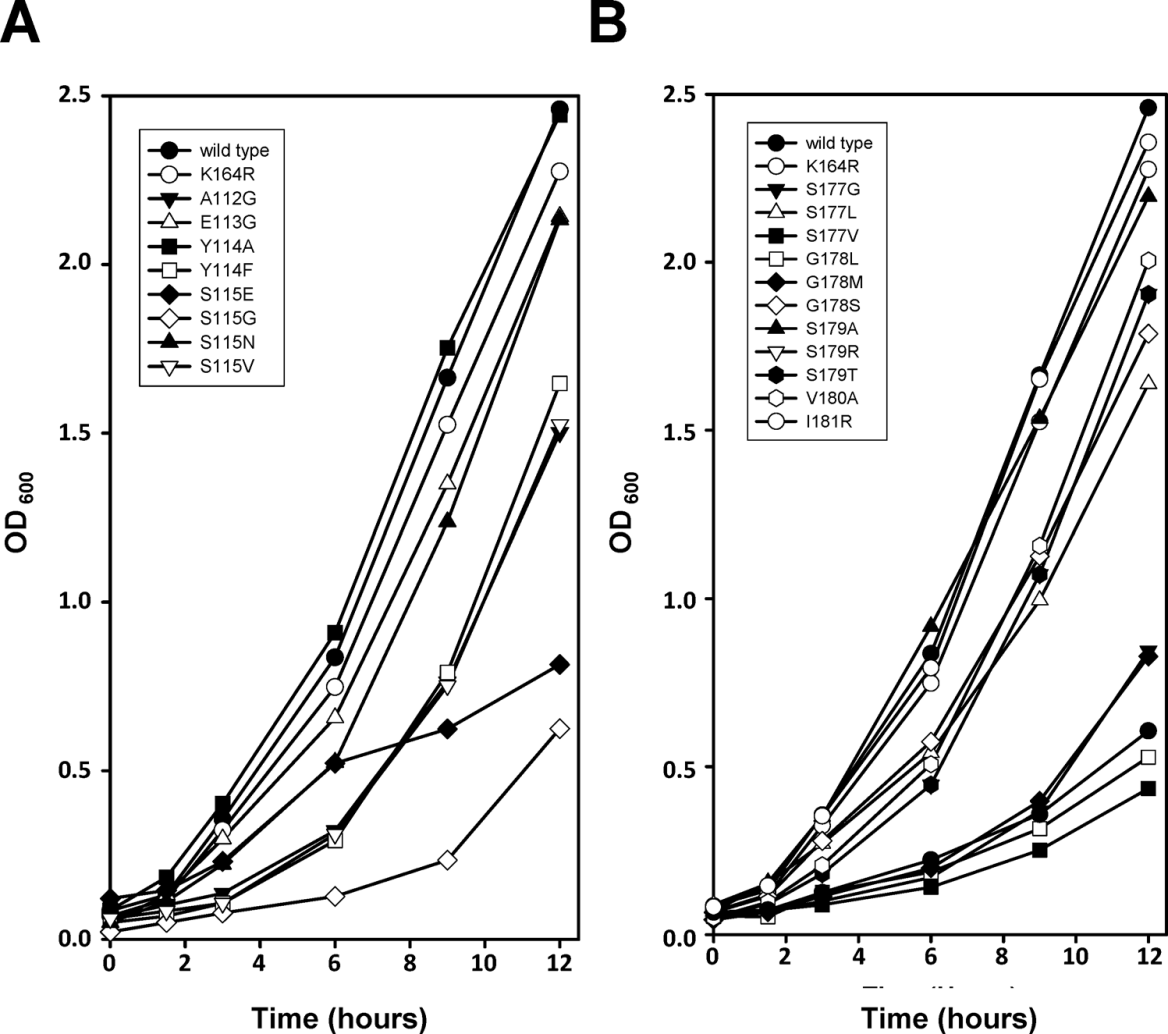
Growth rates of PCNA mutants in liquid culture. Yeast cultures expressing either wild type PCNA or mutant PCNA were grown in liquid culture to an OD_600_ readings were taken at 0, 1.5, 3, 6, 9, and 12 hours. Growth curves for strains expressing mutants with substitutions in β strand I_1_ **(A)** and strains expressing mutants with substitutions in β strand D_2_ **(B)** were plotted along with wild type and K164R mutant PCNA. Experiments were performed in duplicate and average values are reported.

We first measured the growth rate of these 17 strains in liquid media (Fig 3). Twelve of these strains (A112G, Y114F, S115E, S115G, S115V, S177G, S177L, S177V, G178L, G178M, S178T, and V180A) had slower growth rates than the wild type strain of approximately 4 to 5 hours. The remaining five strains had growth rates similar to the wild type strain of approximately 2 to 3 hours. These data agree reasonably well with the qualitative spotting assay described above in which the A112G, S177G and G178M strains showed minor defects in cell growth and the S115E and G178L strain showed a major defect in cell growth. The Y114F, S115V, and V180A mutants, which had a slower growth rate in this assay, showed no growth defect in the spotting assay.

**Fig 4:**
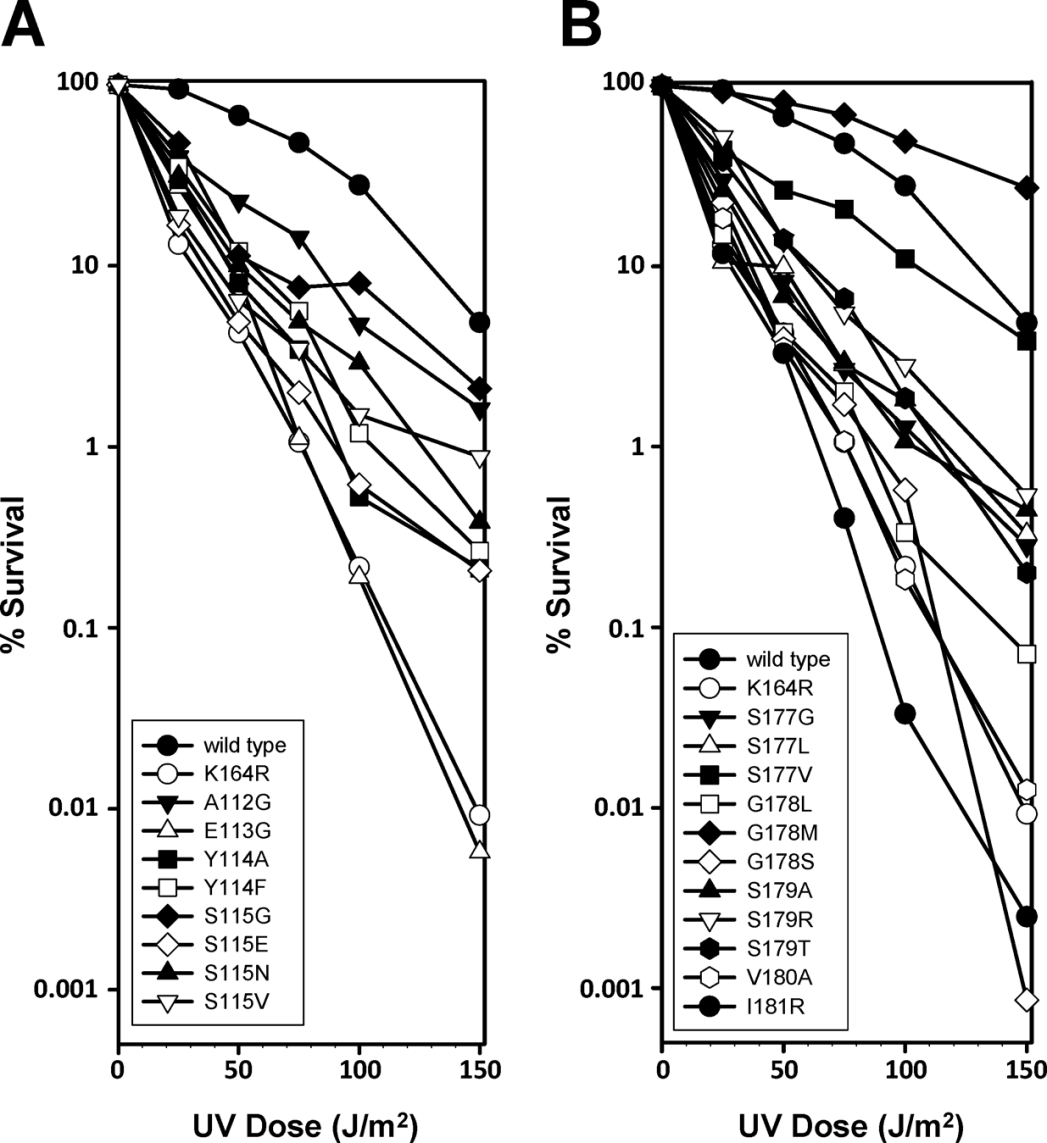
UV sensitivity of the PCNA mutants. Percent survival was plotted as a function of UV dose for strains expressing the β strand I_1_ PCNA mutants **(A)** and the D_2_ PCNA mutants **(B)**. Also included were strains expressing wild type, K164R, E113G, and G178S PCNA. Experiments were repeated in triplicate and average values are reported.

We next measured the UV sensitivity of these 17 strains (Fig 4). As a negative control, we included a strain producing the K164R mutant protein. Because the previously identified E113G and G178S mutants are known to be sensitive to UV radiation, we also included them for comparison. All 17 strains had increased sensitivities to UV radiation to some extent compared to the wild type except G178M. Six of these strains (Y114A, Y114F, S115E, G178L, V180A, and I181R) had the same increased level of UV sensitivity as the K164R control strain. The other ten strains had sensitivities to UV radiation in-between the wild type and the K164R control. While these data generally agree with the qualitative spotting assay described above, they provide a more rigorous, quantitative measure of the UV sensitivities of these strains and thus a more straightforward comparison.

**Fig 5:**
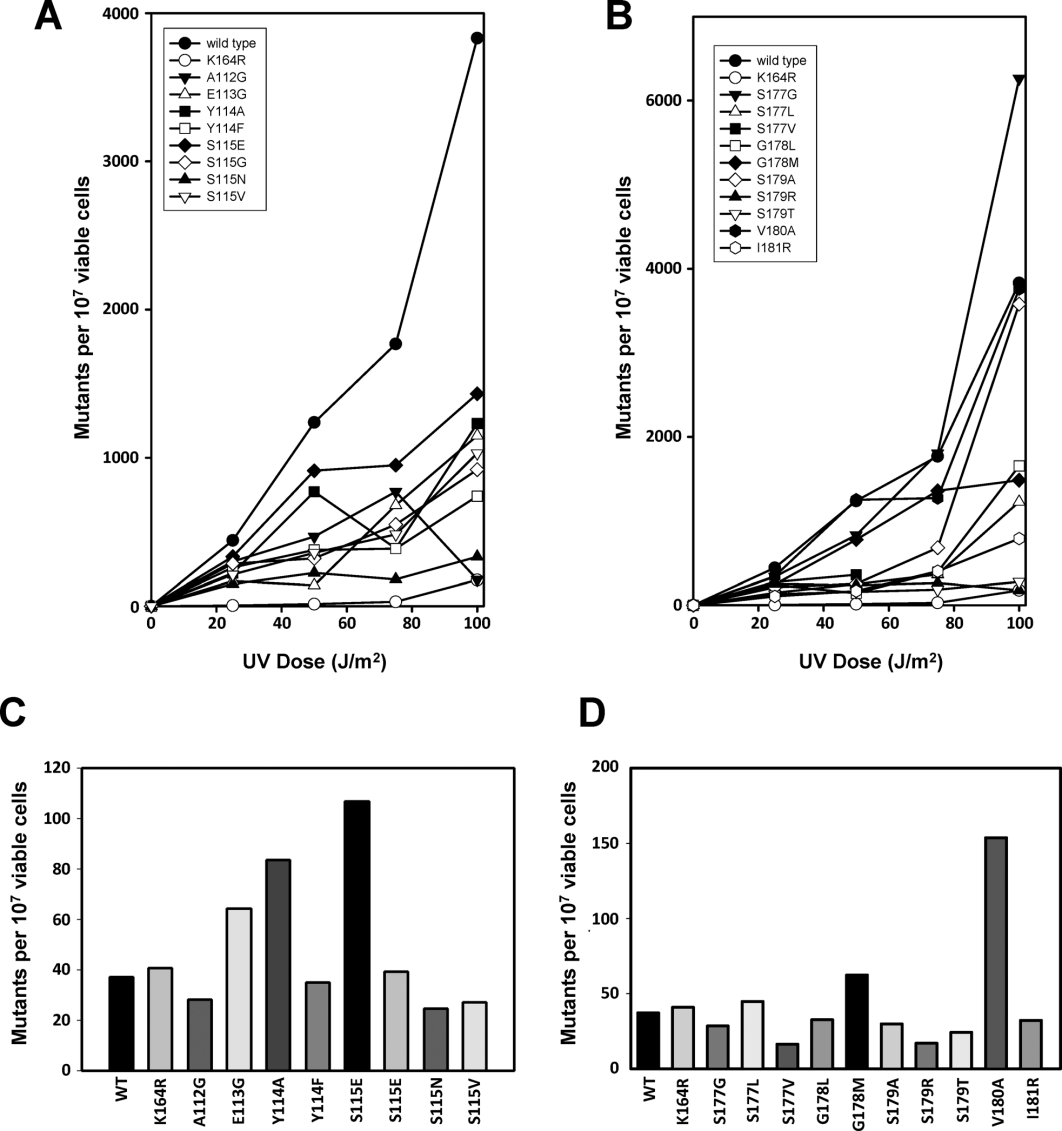
Spontaneous and UV-induced mutagenesis of the PCNA mutants. The number of UV-induced *CAN1*^*S*^ to *can1*^*r*^ mutants was plotted as a function of UV dose for strains expressing the β strand I_1_ PCNA mutants **(A)** and the D_2_ PCNA mutants **(B)**. Also included were strains expressing the wild type, K164R, E113G, and G178S PCNA. The numbers of spontaneous *CAN1*^*S*^ to *can1*^*r*^ mutants are shown for strains expressing the β strand I_1_ PCNA mutants **(C)** and the D_2_ PCNA mutants **(D)**. Experiments were repeated in triplicate and average values are reported.

We then examined the rates of spontaneous mutagenesis and the rates of UV-induced mutagenesis of these 17 strains as well as E113G and G178S (Fig 5). Only six strains had an increase in the rate of spontaneous mutagenesis compared to the wild type. The E113G, Y114A, and G178M strains had an approximately two-fold increase in the rate of spontaneous mutagenesis, while the S115E and V180A strains had an approximately 4-fold increase. By contrast, all 17 strains, except the S177G, S179A, and V180A mutant strains, had statistically significant defects in the rate of UV-induced mutagenesis (with p values less than 0.05). Five of these strains (S115N, S177V, S179R, S179T, and I181R) had complete defects in UV-induced mutagenesis similar to that observed with the K164R control strain. The other nine strains (A112G, Y114A, Y114F, S115E, S115G, S115V, S177L, G178L, and G178M) had partial defects in UV-induced mutagenesis that are more pronounced at lower doses of UV radiation. A defect in UV-induced mutagenesis is a characteristic indicator of a defect in error-prone TLS. These results are all summarized in **Table 2**.

**Table 2:** Summary of phenotypes of PCNA mutants.

	Cell growth	UV Resistance	UV-induced Mutagenesis
Wild type	+++	+++	+++
K164R	+++	-	-
A112G	+	+	+
Y114A	+++	-	+
Y114F	+	-	+
S115E	-	-	+
S115G	-	+	+
S115N	+++	+	-
S115V	+	+	+
S177G	-	+	+++
S177L	+	+	+
S177V	-	+	-
G178L	-	-	+
G178M	-	+++	+
S179A	+++	+	+++
S179R	+++	+	-
S179T	+	+	-
V180V	+	-	+++
I181R	+++	-	-

The descriptions of these phenotypes are given in the text.

## Discussion

Two substitutions at the subunit interface of PCNA have been previously shown to cause defects in TLS [20–22]. The E113G substitution is located in β strand I_1_ of domain 1, and the G178S substitution is located in β strand D_2_ of domain 2. Cells producing these proteins are unable to carry out error-prone TLS and therefore have significantly reduced rates of UV-induced mutagenesis. In addition, both of these mutant proteins have similar structural alterations in these β strands and in loop J (residues 105 to 108), which leads into strand I_1_. Moreover, both mutant proteins form less stable trimers than does the wild type PCNA protein. It has been argued that the structural changes seen at the subunit interface and the concomitant decrease in trimer stability are the bases for the defect in TLS observed with these two mutant proteins [23,25].

Analyses of X-ray crystal structures of these two mutant proteins have suggested that the observed structural changes at the subunit interface may be peculiar to these specific substitutions. In the case of the E113G mutant protein, it has been argued that the additional backbone flexibility in β strand I_1_ resulting from the substitution allows loop J to adopt an aberrant, but more energetically favorable conformation than it would in the wild type protein [26]. In the case of the G178S mutant protein, it has been suggested that an additional hydrogen bond between the hydroxyl group on the substituted serine side chain and the backbone carbonyl oxygen of residue 113 alters the trajectory of β strand I_1_ leading to an alteration of the conformation of loop J [23].

In the present study, the β strands that comprise the subunit interface of PCNA were randomly mutated to determine the effect of these mutations on PCNA’s ability to stimulate error-prone TLS. Based on these structural considerations above, we did not expect a wide range of substitutions at the PCNA subunit interface to lead to defects in TLS. Surprisingly we found that of the subset of 17 substitutions whose UV sensitivities and mutagenesis rates we studied quantitatively, 14 (82% of them) had a partial or complete reduction in the rate of UV-induced mutagenesis. Four of these substitutions have a complete reduction in the rate UV-induced mutagenesis (A112G, S115N, S179R, and S179T) and ten have a partial reduction (Y114A, Y114F, S115E, S115G, S115V, S177L, S177V, G178L, G178M, and I181R). This phenotype is the hallmark of a defect in error-prone TLS, and thus we have identified fourteen new substitutions in PCNA that cause a partial or complete defect in TLS.

These results demonstrate that a large proportion of random mutations at the PCNA subunit interface cause a defect in TLS. Moreover, there does not appear to be any obvious trend with respect to the position and nature of the TLS-defective mutations. It is likely that these newly identified mutations at the PCNA subunit interface affect trimer stability and altered position of loop J in a similar manner as seen with the E113G and G178S mutant proteins. Further studies of the biochemical and biophysical properties of some of these newly identified PCNA mutant proteins will be needed to fully understand why some mutant proteins are partially defective in TLS and why others are completely defective in TLS. In addition, continued studies of this set of PCNA mutant proteins–including determinations of their X-ray crystal structures, trimer stabilities, and abilities to support the activity of translesion synthesis polymerases–will provide important insights into the role of PCNA in TLS and into novel strategies to inhibit error-prone TLS.

## Abbreviations

dNTP: deoxynucleoside triphosphate
IDCL: inter-domain connector loop
PAGE: polyacrylamide gel electrophoresis
PCNA: proliferating cell nuclear antigen
PIP: PCNA-interacting protein
pol: polymerase
MMR: mismatch repair
RFC: replication factor C
TLS: translesion synthesis
UV: ultraviolet
